# A nomogram combining plasma fibrinogen and systemic immune‑inflammation index predicts survival in patients with resectable gastric cancer

**DOI:** 10.1038/s41598-021-89648-9

**Published:** 2021-05-13

**Authors:** Pan-Xing Wang, Hai-Jiang Wang, Jia-Huang Liu, Guang-Lin Qiu, Jing Lu, Lin Fan, Xin-Hua Liao, Xiang-Ming Che

**Affiliations:** Department of General Surgery, The First Affiliated Hospital of Xi’an Jiaotong University, 277 Yanta West Road, Xi’an, 710061 Shanxi Province China

**Keywords:** Gastrointestinal cancer, Prognostic markers

## Abstract

Hyperfibrinogenemia and cancer-associated systemic inflammatory response are strongly associated with cancer progression and prognosis. We aimed to develop a novel prognostic score (F-SII score) on the basis of preoperative fibrinogen (F) and systemic immunoinflammatory index (SII), and evaluate its predictive value in patients with resectable gastric cancer (GC). Patients diagnosed with GC between January 2012 and December 2016 were reviewed. The F-SII score was 2 for patients with a high fibrinogen level (≥ 3.37 g/L) and a high SII (≥ 372.8), whereas that for patients with one or neither was 1 or 0, respectively. A high F-SII score was significantly associated with older patient age, a high ASA score, large tumor size, large proportion of perineural invasion, and late TNM stage. Multivariate analysis indicated that the F-SII score, histological grade, and TNM stage were independent factors for overall survival (OS). The Harrell's concordance index (C-index) of a nomogram based on the F-SII score and several clinicopathological manifestations was 0.72, which showed a better predictive ability for OS than the TNM stage alone (0.68). In conclusion, preoperative F-SII may serve as a useful predictive factor for OS and refine outcome prediction for patients with resectable GC combined with traditional clinicopathological analysis.

## Introduction

Gastric cancer (GC) is the fifth most common malignancy and the third leading cause of cancer-associated mortality worldwide^1^. Despite the significant advances in therapeutic methods, including surgical techniques and adjuvant therapy, the prognosis of GC has not been substantially improved^2^. Therefore, identifying independent prognostic factors is of utmost importance for optimizing personalized treatment.

Inflammation plays a vital role in the tumor microenvironment and is related to the promotion, progression, invasion, and metastasis of tumors^3^. Pretreatment serum-based inflammatory biomarkers, such as the neutrophil-to-lymphocyte ratio (NLR)^4^, platelet-to-lymphocyte ratio (PLR)^5^, and lymphocyte-to-monocyte ratio (LMR)^6^, was used to predict the prognosis of various tumors. Recently, the systemic immune-inflammation index (SII), a newly emerging prognostic score as an integrated expression of neutrophils, lymphocytes, and platelets, is deemed to provide better prognostic information on patients with hepatocellular carcinoma^7^, pancreatic cancer^8^, germ-cell tumors^9^, and gastric cancer^10^. In addition to inflammatory biomarkers, fibrinogen is a 340-kDa glycoprotein, synthesized as an acute-phase reactant glycoprotein by hepatocytes and has an important role in the coagulation process^11^. Mounting evidence has reported that hyperfibrinogenemia is related to tumor progression, metastasis, and prognosis in patients with gastric cancer^12,13^. High SII and elevated fibrinogen independently predict a worse prognosis of GC. However, the evidence is still limited regarding the joint association between both conditions and the prognosis in patients with GC.

Therefore, in this study, we developed a novel prognostic score combining the fibrinogen (F) and systemic immune-inflammation index (F-SII) score, and evaluated the association between the F-SII score and the prognosis in patients with resectable GC. Moreover, a nomogram combining the F-SII score with TNM stage, and histological grade, was established to predict 3- and 5-yr overall survival (OS) for patients with resectable GC.

## Results

### Patient characteristics

In the present study, a total of 608 patients were included. The median patient age was 61 years (range 25–86 years). The median follow-up period was 56.0 months (interquartile range, 41–71 months). The 1-yr, 3-yr, and 5-yr OS rates for the present study were 85.9%, 58.3%, and 48.0%, respectively. The baseline characteristics of the patients are summarized in Table 1.

**Table 1:** Patient and tumour characteristics.

Characteristics	No	%
**Age (years)**		
≥ 60	354	58.2
< 60	254	41.8
**Sex**		
Male	461	75.8
Female	147	24.2
**BMI (kg/m**^**2**^**)**		
≥ 24	160	26.3
< 24	448	73.7
**ASA score**		
1	40	6.6
2	446	73.4
3	122	20.1
**Tumor location**		
Upper	207	34.0
Middle	124	20.4
Lower	277	45.6
**Tumor size (cm**)		
≥ 5	267	43.9
< 5	341	56.1
**Histological grade**		
Well or moderately differentiated	202	33.2
Poorly or not differentiated	406	66.8
**Vascular invasion**		
Yes	68	11.2
No	540	88.8
**Perineural invasion**		
Yes	217	35.7
No	391	64.3
**Lymphatic invasion**		
Yes	65	10.7
No	543	89.3
**Pathological tumor stage**		
T1	98	16.1
T2	36	5.9
T3	47	7.7
T4	427	70.2
**Pathological lymph node stage**		
N0	225	37
N1	105	17.3
N2	117	19.2
N3	161	26.5
**TNM stage**		
I	115	18.9
II	141	23.2
III	352	57.9
**Adjuvant chemotherapy**		
Yes	325	53.5
No	283	46.5
**Fibrinogen level (g/L)**		
≥ 3.37	258	42.4
< 3.37	350	57.6
**SII**		
≥ 372.8	328	53.9
< 372.8	280	46.1
**F-SII score**		
0	208	34.2
1	214	35.2
2	186	30.6

*BMI* Body Mass Index, *ASA score* American Society of Anesthesiologists score, *SII* Systemic immune-inflammation index, *F-SII* Fibrinogen and systemic immune-inflammation index.

### Associations of the plasma fibrinogen level, SII, and F-SII score

The univariate analysis showed that the fibrinogen level and SII were associated with OS. Age, ASA score, tumor location, tumor size, histological grade, perineural invasion, and TNM stage also had a significant effect on OS (Table 2). According to our multivariate analysis, the fibrinogen level and SII were independent factors for prognosis (HR, 1.509; 95% CI, 1.181–1.929; *P* = 0.001; HR, 1.452; 95% CI, 1.128–1.868; *P* = 0.004, respectively). In addition, well or moderately differentiated tumors and stage I disease were associated with good prognosis in GC (Table 2).

**Table 2:** Univariate and multivariate Cox regression analyses for overall survival in patients with gastric cancer.

Characteristics	Univariate analysis	*P*-values	Multivariate analysis^a^	*P*-values	Multivariate analysis^b^	*P*-values
	HR (95%CI)		HR (95%CI)		HR (95%CI)	
Age (≥ 60 vs. < 60 years)	1.269 (1.006–1.602)	0.045	1.052 (0.821–1.348)	0.689	1.054 (0.823–1.349)	0.678
Sex (Female vs. Male)	0.815 (0.615–1.073)	0.145				
BMI (≥ 24 vs. < 24 kg/m2)	0.895 (0.689–1.162)	0.405				
**ASA score**		0.009		0.272		0.282
2 vs. 1	1.118 (0.690–1.813)	0.650	0.993 (0.608–1.623)	0.979	0.992 (0.607–1.620)	0.973
3 vs. 1	1.663 (0.992–2.786)	0.054	1.240 (0.728–2.114)	0.428	1.235 (0.725–2.106)	0.437
**Tumor location**		0.004		0.227		0.224
Middle vs. upper	0.825 (0.606–1.124)	0.224	0.984 (0.713–1.358)	0.923	0.986 (0.714–1.362)	0.933
Lower vs. upper	0.651 (0.505–0.840)	0.001	0.810 (0.624–1.051)	0.112	0.809 (0.624–1.050)	0.112
Tumor size (≥ 5 vs. < 5 cm)	2.187 (1.737–2.753)	< 0.001	1.251 (0.983–1.591)	0.069	1.253 (0.985–1.594)	0.066
Histological grade: Well or moderately differentiated vs. Poorly or not differentiated	0.498 (0.382–0.651)	< 0.001	0.721 (0.546–0.952)	0.021	0.721 (0.546–0.952)	0.021
Vascular invasion (Yes vs. No)	1.309 (0.938–1.828)	0.113				
Perineural invasion (Yes vs. No)	1.714 (1.362–2.157)	< 0.001	1.034 (0.815–1.312)	0.781	1.033 (0.813–1.311)	0.792
Lymphatic invasion (Yes vs. No)	1.159 (0.817–1.643)	0.408				
**TNM stage**		< 0.001		< 0.001		< 0.001
II vs. I	4.254 (2.212–8.183)	< 0.001	3.125 (1.602–6.094)	0.001	3.119 (1.599–6.084)	0.001
III vs. I	11.485 (6.267–21.048)	< 0.001	7.619 (4.050–14.332)	< 0.001	7.614 (4.047–14.322)	< 0.001
Adjuvant chemotherapy (Yes vs. No)	1.033 (0.823–1.297)	0.780				
Fibrinogen level (≥ 3.37 vs. < 3.37 g/L)	2.097 (1.668–2.636)	< 0.001	1.509 (1.181–1.929)	0.001		
SII (≥ 372.8 vs. < 372.8)	2.013 (1.584–2.558)	< 0.001	1.452 (1.128–1.868)	0.004		
**F-SII score**		< 0.001				< 0.001
1 vs. 0	1.493 (1.094–2.036)	0.012			1.505 (1.097–2.066)	0.011
2 vs. 0	2.656 (1.979–3.564)	< 0.001			2.201 (1.612–3.004)	< 0.001

*BMI* Body Mass Index, *ASA score* American Society of Anesthesiologists score, *SII* Systemic immune‑inflammation index, *F-SII score* Fibrinogen and systemic immune‑inflammation index score.

^a^Adjustment for all variables listed in the table, except for sex, BMI, vascular invasion, lymphatic invasion, chemotherapy, and F-SII score.

^b^Adjustment for all variables listed in the table, except for sex, BMI, vascular invasion, lymphatic invasion, chemotherapy, fibrinogen level, and SII.

**Figure 1:**
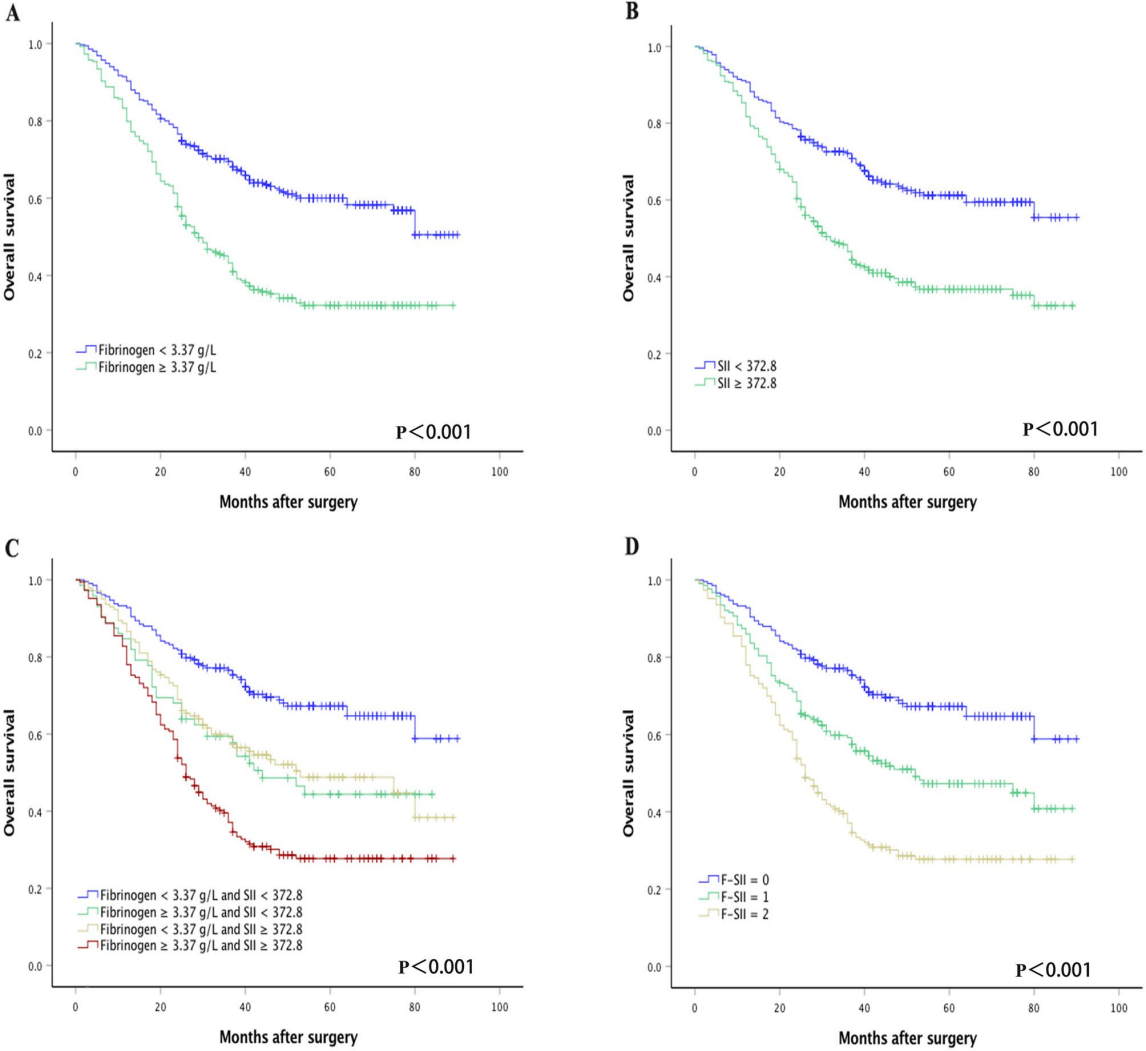
Kaplan–Meier analysis for OS of GC patients according to preoperative plasma fibrinogen level and the systemic immune-inflammation index (SII). Kaplan–Meier analysis for OS according to (**A**) preoperative plasma fibrinogen level, (**B**) preoperative SII, (**C**) combination of preoperative plasma fibrinogen and SII, and (**D**) F-SII score.

Patients were classified into two independent groups based on the cutoff thresholds of fibrinogen and SII (low < 3.37 g/L or high ≥ 3.37 g/L and low < 372.8 or high ≥ 372.8, respectively) for subsequent analyses. It is showed that decreased plasma fibrinogen and SII were both associated with shorter OS (both *P* < 0.001) (Fig. 1A,B). To further investigate the effect of the plasma fibrinogen level and SII on patient prognosis, we divided the patients into four groups based on the cutoff thresholds of fibrinogen and SII. Kaplan–Meier analysis indicated obvious differences between the four subgroups (*P* < 0.001, Fig. 1C). There was no significant difference in subgroups of either plasma fibrinogen ≥ 3.37 g/L or SII ≥ 372.8 (*P* > 0.05, Fig. 1C). Therefore, we combined the two subgroups. Patients were divided into three F-SII score subgroups based on the following criteria: score 2, both a high fibrinogen level (≥ 3.37 g/L) and a high SII (≥ 372.8); score 1, either a high fibrinogen level or a high SII; and score 0, both a low fibrinogen level (< 3.37 g/L) and a low SII (< 372.8).

### The F-SII score independently predicts OS

**Figure 2:**
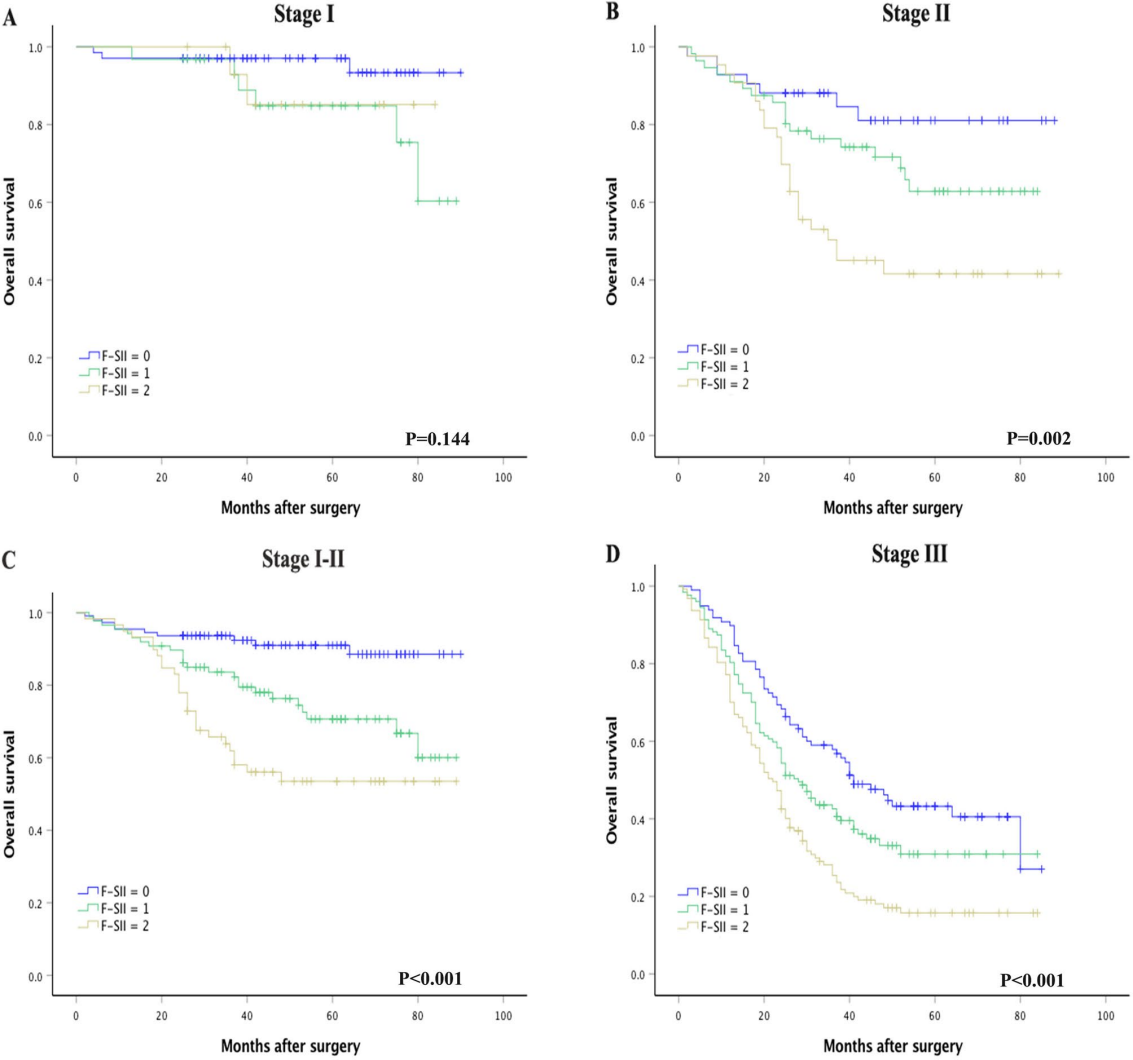
Kaplan–Meier analysis of OS of GC patients at each TNM stage according to the F-SII score. (**A**) Association of the F-SII score with the OS of patients with stage I GC. (**B**) Association of the F-SII score with the OS of patients with stage II GC. (**C**) Association of the F-SII score with the OS of patients with stage I-II GC. (**D**) Association of the F-SII score with the OS of patients with stage III GC.

**Figure 3:**
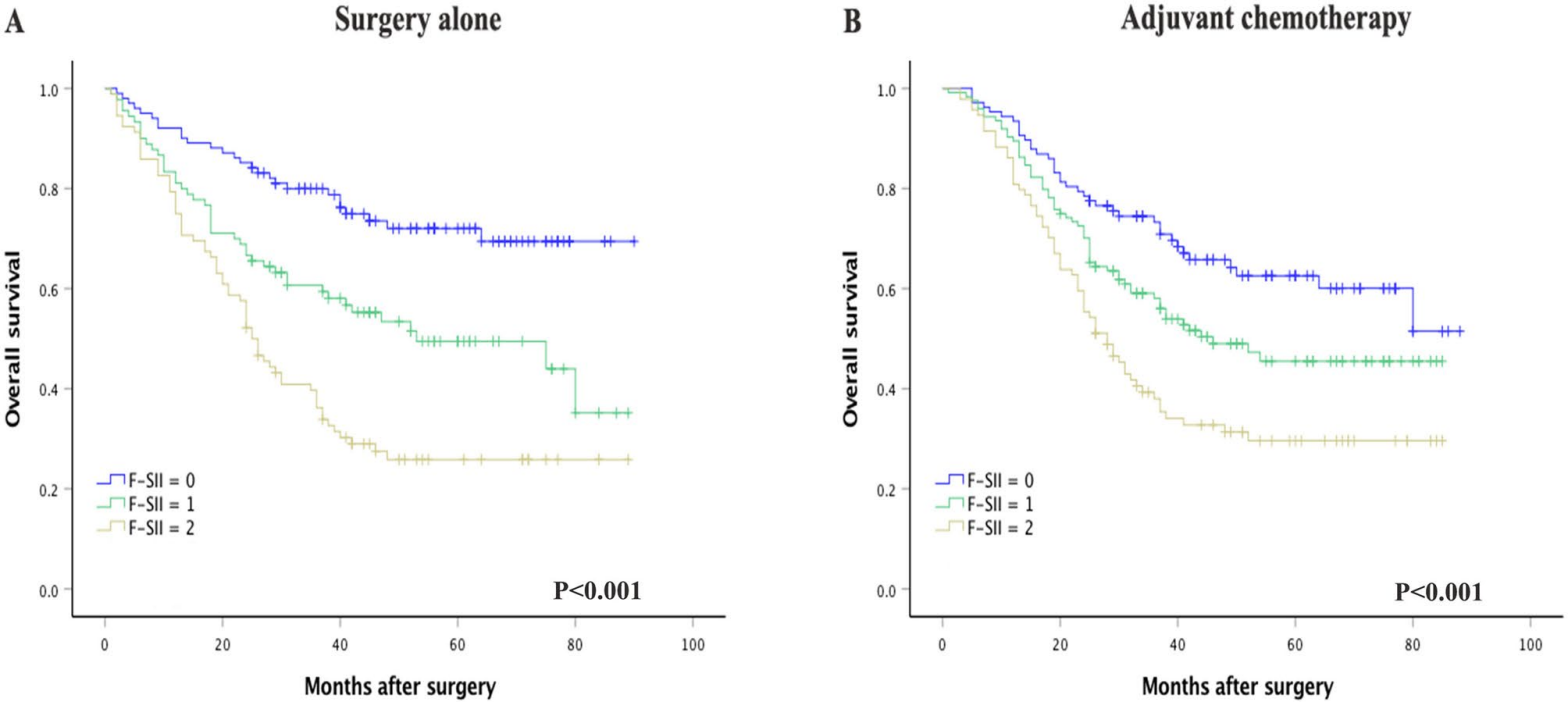
(**A**) Association of the F-SII score with the OS in the surgery alone group. (**B**) Association of the F-SII score with the OS in the adjuvant chemotherapy group.

The univariate analysis showed that the F-SII score had a significant effect on OS (*P* < 0.001). The results of the multivariate analysis indicated that the F-SII score, histological grade, and TNM stage were independent prognostic factors of OS in GC patients (all *P* < 0.05) (Table 2). Kaplan–Meier analysis showed that a high F-SII score was associated with short OS (*P* < 0.05, Fig. 1D). To further analyze the F-SII score's performance in patients with different TNM stages and adjuvant chemotherapy strategies, we conducted a subgroup analysis. When stratified by TNM stage, there was no significant difference in 5-yr OS between the three groups of patients with stage I GC (*P* = 0.144; Fig. 2A). However, the prognostic value of the F-SII score was maintained for stages II (*P* = 0.002; Fig. 2B), I–II (*P* < 0.001; Fig. 2C) and III (*P* < 0.001; Fig. 2D). The F-SII also stratified OS irrespective of adjuvant chemotherapy administration (*P* < 0.05; Fig. 3A,B).

### Associations of the plasma fibrinogen level, SII, and F-SII score with clinicopathological characteristics

The associations of the plasma fibrinogen level and SII with clinicopathological characteristics are shown in Table 3. Elevated plasma fibrinogen levels and a high SII were associated with older age at surgery (*P* < 0.001 and *P* = 0.021), a high ASA score (*P* = 0.006 and *P* = 0.015), tumor size ≥ 5 cm (both *P* < 0.001), and a late TNM stage (both *P* < 0.001). Moreover, we assessed the association between the F-SII score and clinicopathological factors (Table 3). A high F-SII score was associated with older patient age (*P* < 0.001), a high ASA score (*P* = 0.002), large tumor size (*P* < 0.001), a large proportion of perineural invasion (*P* = 0.033), and late TNM stage (*P* < 0.001) (Table 3).

**Table 3:** Associations of Fibrinogen, SII, and F-SII score with clinicopathological characteristics.

	Fibrinogen level (g/L)			SII			F-SII score			
	< 3.37	≥ 3.37	*P*-values	< 372.8	≥ 372.8	*P*-values	0	1	2	*P*-values
Characteristics	n = 350	n = 258		n = 280	n = 328		n = 208	n = 214	n = 186	
**Age (years)**			< 0.001			0.021				< 0.001
≥ 60	179	175		149	205		101	126	127	
< 60	171	83		131	123		107	88	59	
**Sex**			0.144			0.165				0.904
Male	273	188		205	256		159	160	142	
Female	77	70		75	72		49	54	44	
**BMI (kg/m**^**2**^**)**			0.065			0.124				0.114
≥ 24	102	58		82	78		64	56	40	
< 24	248	200		198	250		144	158	146	
**ASA score**			0.006			0.015				0.002
1	22	18		19	21		14	13	13	
2	273	173		219	227		171	150	125	
3	55	67		42	80		23	51	48	
**Tumor location**			0.559			0.792				0.707
Upper	115	92		96	111		67	77	63	
Middle	69	55		60	64		46	37	41	
Lower	166	111		124	153		95	100	82	
**Tumor size (cm)**			< 0.001			< 0.001				< 0.001
≥ 5	117	150		97	170		60	94	113	
< 5	233	108		183	158		148	120	73	
**Histological grade**			0.179			0.168				0.091
Well or moderately different	124	78		101	101		81	63	58	
Poorly or not differentiated	226	180		179	227		127	151	128	
**Vascular invasion**			0.281			0.55				0.586
Yes	35	33		29	39		20	24	24	
No	315	225		251	289		188	190	162	
**Perineural invasion**			0.236			0.063				0.033
Yes	118	99		89	128		60	87	70	
No	232	159		191	200		148	127	116	
**Lymphatic invasion**			0.707			0.611				0.605
Yes	36	29		28	37		19	26	20	
No	314	229		252	291		189	188	166	
**TNM stage**			< 0.001			< 0.001				< 0.001
I	89	26		78	37		68	31	16	
II	80	61		60	81		42	56	43	
III	181	171		142	210		98	127	127	
**Adjuvant chemotherapy**			0.753			0.913				0.258
Yes	189	136		149	176		107	124	94	
No	161	122		131	152		101	90	92	

*BMI* Body Mass Index, *ASA score* American Society of Anesthesiologists score, *SII* Systemic immune-inflammation index, *F-SII* Fibrinogen and systemic immune-inflammation index.

### Predictive nomogram for OS

**Figure 4:**
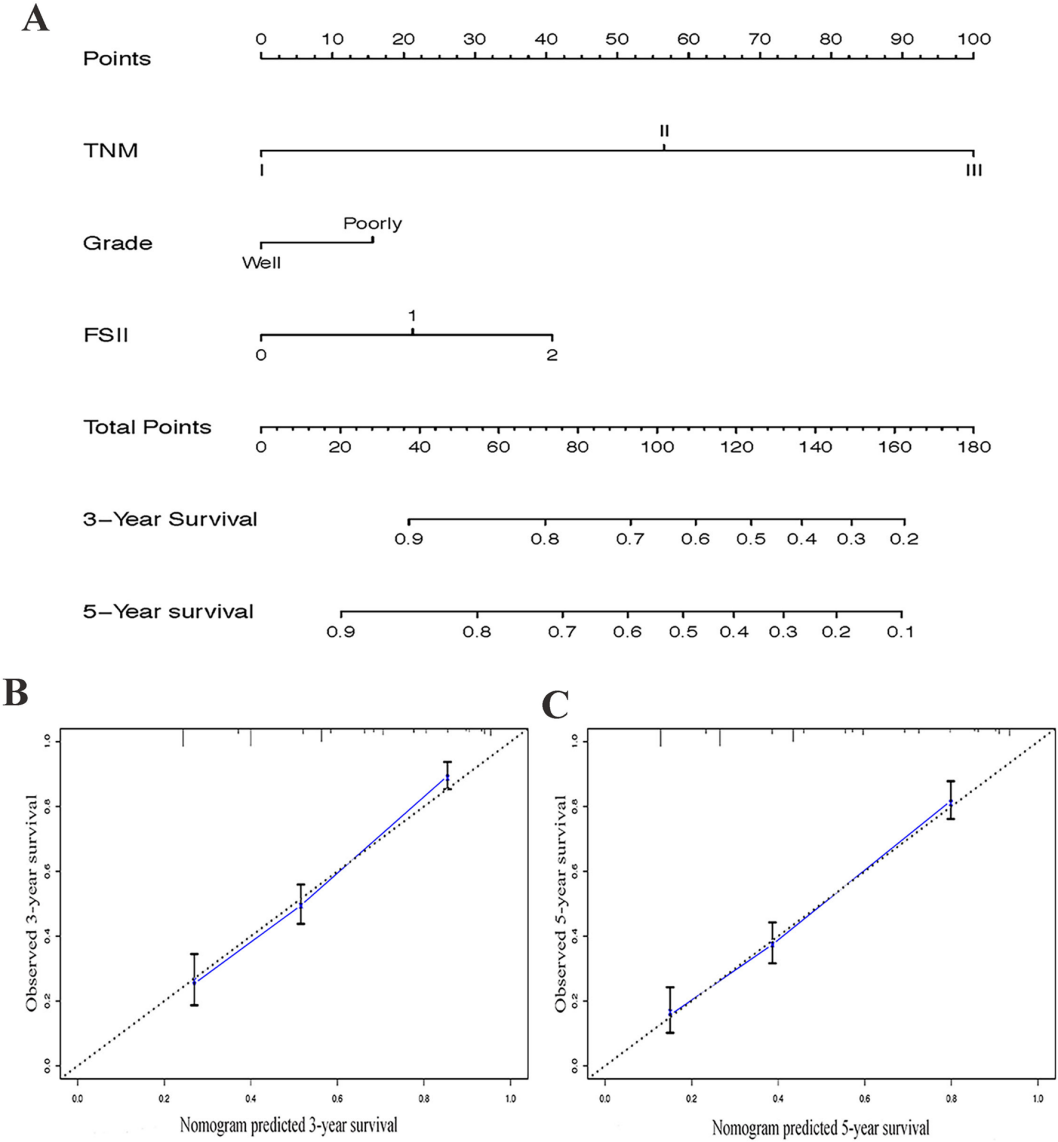
Nomogram for predicting 3- and 5-year OS of GC patients after surgery. (**A**) Nomogram for predicting 3- and 5-year OS of GC patients after surgery. Calibration plot of the nomogram for (**B**) 3-year and (**C**) 5-year survival. The dashed line represents the performance of an ideal nomogram. The blue line indicates the performance of the proposed nomogram. Blue circles are sub-cohorts of the data set; X is the bootstrapped corrected estimate of nomogram with 200 resamples. Vertical bars represent 95% CI. It seems that the nomogram predicts accurately 3- and 5-year OS.

**Figure 5:**
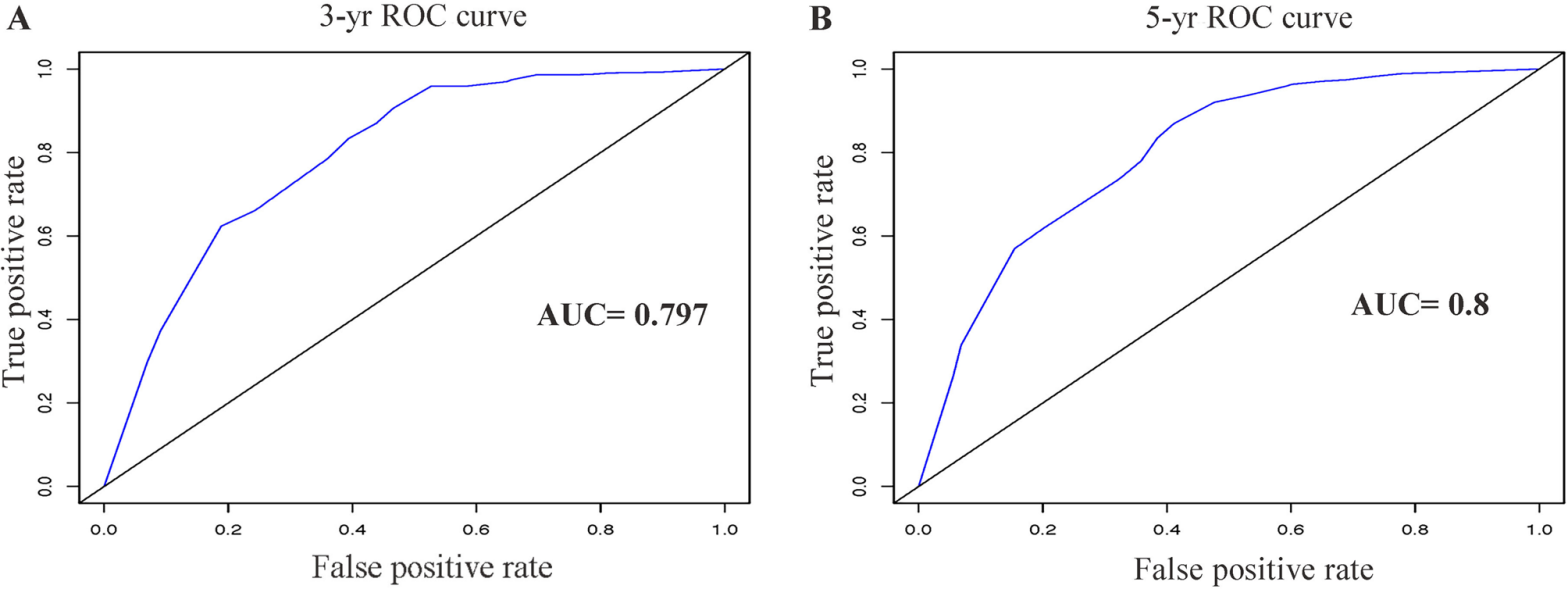
Time-dependent receiver operating characteristic curve analysis for the sensitivity and specificity of the nomograms. Receiver operating characteristic of the nomogram for (**A**) 3-yr survival and (**B**) 5-yr survival.

To evaluate the predictive value of the F-SII score, we constructed a nomogram that integrated the independent prognostic factors consisting of TNM stage, histological grade, and F-SII score (Fig. 4A). In this nomogram, each factor was ascribed a weighted point total that indicated a survival prognosis. For internal validation, the calibration curve suggested that the 3- and 5-yr survival rates predicted by the nomogram were consistent with the actual survival rates (Fig. 4B,C). The Harrell's concordance index (C-index) of the nomogram was 0.72, which showed a better predictive ability for OS than the TNM stage (C-index 0.68) and F-SII (C-index 0.62). The areas under the 3-yr and 5-yr ROC curves of the nomogram were 0.797 and 0.80, respectively (Fig. 5A,B). Therefore, combined with the above results, the nomogram is superior to the TNM staging system in predicting the OS of patients with GC.

## Discussion

In this study, we confirmed that the preoperative plasma fibrinogen and SII were independent prognostic factors in patients with resectable GC. Moreover, the F-SII score, a newly proposed cumulative score, remained an independent prognostic factor in the multivariate analysis. In addition, its prognostic significance was maintained in the subgroup analysis of patients diagnosed with TNM stages I–II or stage III, as well as patients who did or did not receive adjuvant chemotherapy. We found that a high F-SII score was also associated with older age at surgery, a high ASA score, a large tumor size, the presence of perineural invasion, and a late TNM stage. Then, we developed a prognostic nomogram that included the TNM stage, histological grade, and F-SII score and predicted OS with an accuracy of 0.72. Thus, the F-SII score as an easy and inexpensive indicator might provide important prognostic information to help clinicians estimate the patient outcome by combining with conventional clinicopathological analysis. To the best of our knowledge, this is the first study to determine the clinical value of the F-SII score in patients with resectable GC.

It was reported that plasma fibrinogen is synthesized as an acute-phase reactant glycoprotein by hepatocytes. Several studies have reported the mechanisms of hyperfibrinogenemia in various tumors^13–16^. In patients with lung cancer, interleukin-6 produced by tumor cells stimulates the secretion of fibrinogen^15^. More importantly, fibrinogen, which is synthesized by cancer cells, promotes the proliferation of fibroblast growth factor-2^16^. Finally, plasma fibrinogen promotes tumor cell growth and angiogenesis by interacting with fibroblast growth factor-2 and vascular endothelial growth factor^16,17^. On the other hand, since Virchow originally made a link between cancer and inflammation in the nineteenth century, a growing body of evidence^3,18^ has suggested that the levels of inflammatory markers play a vital role in tumor progression and metastasis. Hu et al.^7^ reported that a high SII was related to liver cirrhosis, a large tumor size, low tumor differentiation, early recurrence, high circulating tumor cell levels, and a poor prognosis in patients with hepatocellular carcinoma. Moreover, Wang et al.^10^ found that high SII was associated with old age at surgery, poor Borrmann classification, a large tumor size, advanced tumor invasion, lymph node metastasis, distant metastasis, advanced TNM stage, a high CEA level, and poor outcome in patients with gastric cancer. It was reported that a high SII was also connected with sex, the hemoglobin level, and a poor prognosis in patients with small-cell lung cancer^19^. In the present study, we showed that the plasma fibrinogen level and SII are independent prognostic factors of OS in GC patients. Therefore, we created the F-SII score consisting of the plasma fibrinogen level and SII.

In agreement with previous findings, we demonstrated that both a high fibrinogen level and a high SII (F-SII score 2) are related to advanced tumor stage and a poor prognosis. In contrast, decreased levels of both (F-SII score 0) are related to early tumor stage and a favorable prognosis. Furthermore, a high F-SII score was associated with aggressive tumor biological phenotypes, such as large tumor size, the presence of perineural invasion, and advanced tumor stage. Combined with the above results, the complex interaction between an elevated systemic inflammatory response and tumor progression was partially revealed. Of note, its prognostic significance was still maintained in the subgroup analysis of patients diagnosed with TNM stages I–II or stage III, as well as patients who did or did not receive adjuvant chemotherapy, suggesting that the F-SII score might provide additional prognostic information as a complement to the complete clinicopathological predictive models. As a result, the F-SII score could be an accurate prognostic indicator.

At present, the nomogram fulfills a necessary role in personalisation of oncological treatments by integrating diverse prognostic and determinant variables to generate the probability of a clinical event^20^. In our study, we developed a nomogram that includes the preoperative TNM stage, histological grade, and F-SII score to improve outcome prediction in GC patients after surgery. We found that the nomogram showed more accurate predictive ability than the TNM stage alone. In addition, the F-SII score can be considered a supplement to physical examinations, such as cross-sectional imaging, endoscopic ultrasonography, and endoscopy, to refine risk stratification in patients with gastric cancer before and after treatment.

The strength of our study is that F-SII score measurements were based on standard laboratory tests of plasma fibrinogen and platelet, neutrophil, and lymphocyte counts, which are routinely used in clinical practice. Nevertheless, our study has certain limitations. First, due to the retrospective nature of the study and the lack of external validation, the prognostic significance of the F-SII score in GC patients remains to be examined prospectively in other populations and larger studies in the future. Second, hematological cell counts may be affected by several factors, though we limited some of the possible confounders. Third, we lacked follow-up information for disease-free survival (DFS), and the application of other survival outcomes may strengthen our findings.

## Conclusion

In conclusion, we created a novel and convenient prognostic score named the F-SII score, which was revealed an independent predictor of survival in patients with resectable GC. The F-SII score may be a useful clinical biomarker for identifying patients at high prognostic risk and planning individualized treatment strategies for GC patients.

## Methods

### Patient characteristics

We collected data from 608 consecutive patients with resectable gastric adenocarcinoma who were treated between January 2012 and December 2016 at the Department of General Surgery, The First Affiliated Hospital of Xi'an Jiaotong University. All patients provided informed consent prior to study participation. This study was approved by the Institutional Review Board of the First Affiliated Hospital of Xi'an Jiaotong University and conducted in compliance with the principles of the Declaration of Helsinki for medical research involving humans. The inclusion criteria of this study were as follows: (1) gastric adenocarcinoma confirmed histopathologically, (2) complete medical records, and (3) underwent radical gastrectomy. The exclusion criteria of this study were as follows: (1) other malignancies, (2) neoadjuvant chemotherapy, (3) metastatic disease, (4) autoimmune or other inflammatory diseases, (5) perioperative mortality, (6) hematological disease, (7) intravenous or arterial embolization within 3 months and (8) continuous anticoagulant therapy. We gathered the following clinical, pathologic, and laboratory data of the patients: age, sex, BMI, American Society of Anesthesiologists (ASA) score, tumor location, tumor size, histological grade, vascular invasion, perineural invasion, lymphatic invasion, TNM stage^21^, adjuvant chemotherapy, fibrinogen, and SII. In our hospital, 5-fluorouracil-based adjuvant chemotherapy is routinely delivered to patients with advanced GC^22^.

### The SII and F-SII score

Preoperative plasma fibrinogen, lymphocyte, neutrophil, and platelet count levels were examined in samples obtained before breakfast within 7 days prior to surgery^23,24^. As defined previously, the SII was defined as follows: SII = platelet count × neutrophil count/lymphocyte count^7^. The optimal cut-off values for plasma fibrinogen (low < 3.37; high ≥ 3.37 g/L) and SII (low < 372.8; high ≥ 372.8) were obtained through ROC curves^25^. The F-SII score was established based on the combination of different plasma fibrinogen levels and SII values.

### Follow‑up

Enrolled patients were prospectively followed-up until June 2019. Patients were routinely followed up every 3 months for the first 2 years after treatment and every 6 months thereafter. Patients evaluations included laboratory tests, a physical examination, multislice computed tomography, and other examinations. OS was defined as the time from the date of surgery to death from any cause or the last follow-up.

### Statistical analysis

Statistical analyses were performed using SPSS software (version 25.0; SPSS Inc., Chicago, IL, USA) and R version 3.6.1 software (http://www.r-project.org/). Extension packages, including "survival", "rms", "foreign", and "survivalROC" were also used. Chi-square tests were performed to analyze categorical variables. Kaplan–Meier survival curves were generated, and the log-rank test was performed to compare survival rates. The best cutoff points of plasma fibrinogen and SII were determined using the Youden index and ROC curves. Multivariate analysis using a Cox proportional hazards regression model was used based on variables with a *P*-value of < 0.05 from the univariate analysis. The nomogram was plotted based on the results of the multivariate analysis. The model's predictive accuracy was estimated by the C-index^26^ and ROC curve analysis. The calibration plots were applied to verify the performance characteristics of the predictive nomogram. The significance level for all statistical tests was set at 0.05, and all tests were 2-sided.

## Data Availability

The datasets generated and/or analysed during the current study are available from the corresponding author on reasonable request.
